# Trends in Research of Prenatal Stress From 2011 to 2021: A Bibliometric Study

**DOI:** 10.3389/fped.2022.846560

**Published:** 2022-07-06

**Authors:** Yankai Dong, Shengquan Chen, Zhifei Wang, Yao Ma, Jinfeng Chen, Ge Li, Jiahao Zhou, Yating Ren, Hengyu Ma, Juanping Xie, Hui Li, Zhongliang Zhu

**Affiliations:** ^1^Key Laboratory of Resource Biology and Biotechnology in Western China, Ministry of Education, College of Life Sciences, Institute of Maternal and Infant Health, Northwest University, Xi'an, China; ^2^Department of Neonatology, The First Affiliated Hospital of Xi'an Jiaotong University, Xi'an, China; ^3^School of Medicine, Qinba Chinese Medicine Resources R&D Center, Ankang University, Ankang, China

**Keywords:** prenatal stress, keyword, trends, bibliometric study, visualization

## Abstract

**Background:**

Maternal stress during pregnancy can raise the risk of mental disorders in offspring. The continuous emergence of clinical concepts and the introduction of new technologies are great challenges. In this study, through bibliometric analysis, the research trends and hotspots on prenatal stress (PS) were explored to comprehend clinical treatments and recommend future scientific research directions.

**Methods:**

Studies on PS published on the Web of Science Core Collection (WoSCC) database between 2011 and 2021 were reviewed. Bibliometric analysis was conducted according to the number of publications, keywords, journals, citations, affiliations, and countries. With the data collected from the WoSCC, visualization of geographic distribution; clustering analysis of keywords, affiliations, and authors; and descriptive analysis and review of PS were carried out.

**Results:**

A total of 7,087 articles published in 2011–2021 were retrieved. During this period, the number of publications increased. Psychoneuroendocrinology is the leading journal on PS. The largest contributor was the United States. The University of California system was leading among institutions conducting relevant research. Wang H, King S, and Tain YL were scholars with significant contributions. Hotspots were classified into four clusters, namely, pregnancy, prenatal stress, oxidative stress, and growth.

**Conclusion:**

The number of studies on PS increased. Journals, countries, institutions, researchers with the most contributions, and most cited articles worldwide were extracted. Studies have mostly concentrated on treating diseases, the application of new technologies, and the analysis of epidemiological characteristics. Multidisciplinary integration is becoming the focus of current development. Epigenetics is increasingly used in studies on PS. Thus, it constitutes a solid foundation for future clinical medical and scientific research.

## Introduction

In a woman's life, pregnancy is an important event (1). Prenatal stress (PS) refers to the exposure of pregnant women to stressors during pregnancy that may indirectly affect the fetus (2). In other words, PS is the stress experienced by pregnant women before giving birth (3). Exposure to adverse stimuli during pregnancy can cause perpetual negative effects on emotion and cognition, thereby increasing the risk of psychiatric disorders in offspring (4, 5). Maternal stress has been described as an important component in the development of offspring's cerebrum, altering the susceptibility to diseases in later life (6). A severe case of PS can lead to lifelong neuropsychiatric complications (7, 8). The consequences of this negative impact can also be passed from generation to generation (9). Maternal PS can be acute, such as by exposure to sudden incidents or other accidents, or chronic and associated with ongoing events (10). Life stress events including work stress, family conflict, and economic difficulties adversely affect women during pregnancy (1). In rodent models, harmful stimulation during pregnancy can induce behavioral changes, including abnormal social behaviors, heightened anxiety-like behavior, and cognitive deficits in offspring (5, 11). Although little is known about it, the complex interaction of the cellular and molecular mechanisms leads to abnormal and pathological results caused by maternal PS. To solve these complex risks, further understanding of the changes in neural development caused by the molecular and cellular mechanisms of the maternal physiological stress response is urgently needed (12). Currently, most reviews on PS have retrospectively analyzed or summarized a few articles (13–15). Key institutions, authors, or publications in this field cannot be found in these reviews, and they fail to expand on topics. Hence, a beginner would spend considerable time and effort reviewing current publications on PS, and predictions of the frontiers in this field are lacking. Accordingly, for researchers, improving the efficiency and guiding studies are important. They must obtain data or information on the latest state of research, measure current practice, and identify gaps. Therefore, solving this series of problems and visualizing the research status, hotspots, and frontiers of PS are imperative.

Bibliometrics, first proposed by Pritchard in 1969, is an integrated analysis method of quantifying the content of the literature (16). As a mature and popular investigation method in information science, bibliometrics is an effective tool to study the discipline status and objectively reflect the discipline development (17). It also plays a major part in predicting development directions and evaluating research value in a certain field (18). Moreover, bibliometrics can effectively provide proof to make funding decisions and implement strategies (19). Bibliometric analysis results have been obtained in many research fields, such as medicine (20), CO_2_ conversion technology (18), epilepsy occurrence, circadian rhythm (21), and berberine extraction (16). Nevertheless, bibliometric research on PS remains rare. Accordingly, the present study aimed to systematically analyze studies on PS and clarify the hotspots and current state in this research field by using CiteSpace (22) and VOSviewer (23).

## Materials and Methods

### Data Sources and Search Strategies

**Figure 1:**
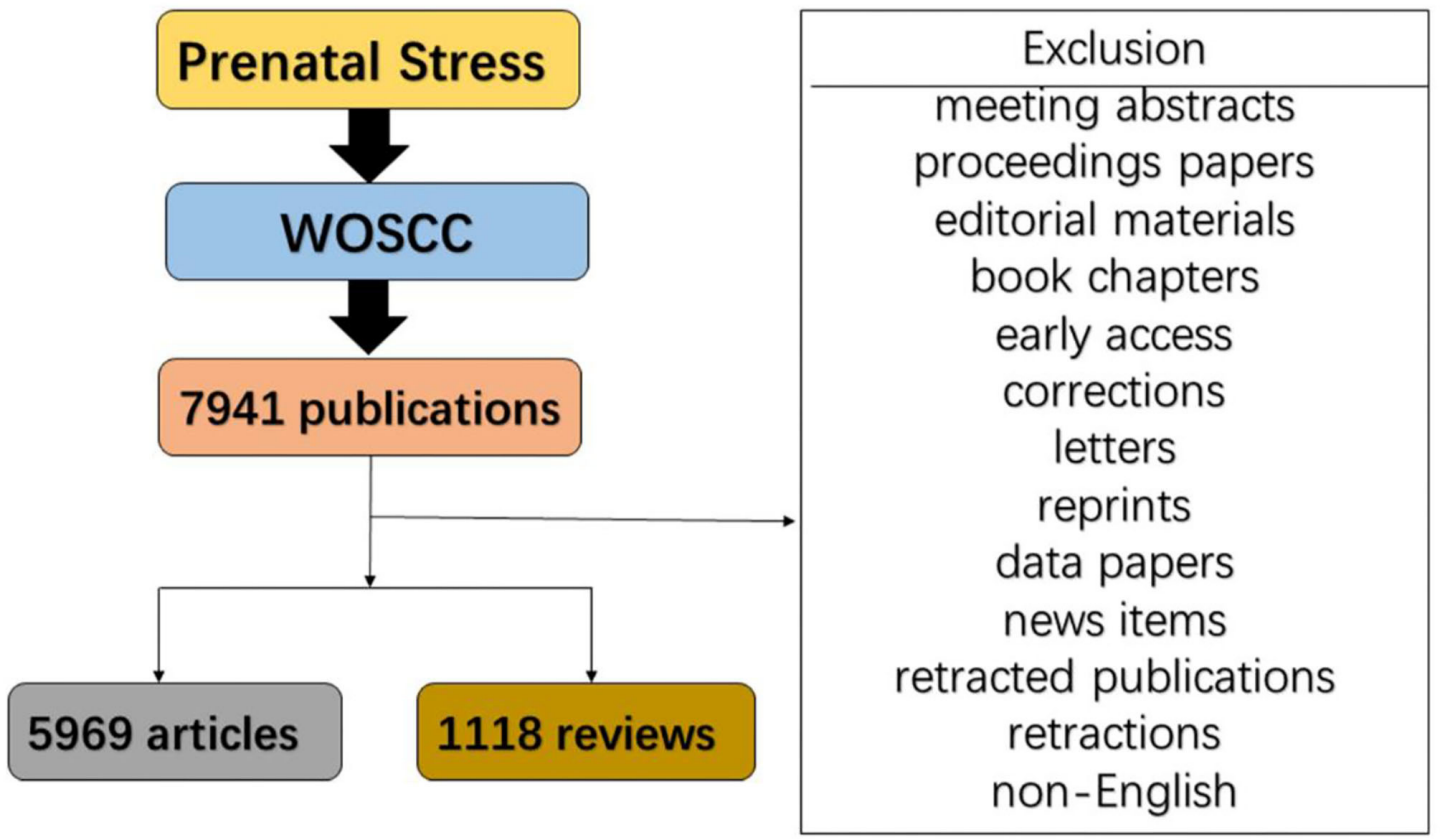
Flowchart of the research.

Web of Science Core Collection (WoSCC) has been employed widely because it can provide a comprehensive and standardized set of data. In this study, the literature dataset was compiled on the basis of WoSCC. Articles were retrieved on the same day (March 7, 2022) to avoid various kinds of deviations because the database is updated rapidly. The timespan of literature retrieval was set between 2011 and 2021 to explore the global scientific trends in PS research. The search term was presented as follows: [TS (Topic)=(prenatal stress)] NOT [TS=(stress in early childhood) OR TS=(paternal pre-conception stress)]. Among various kinds of publications, original articles and reviews which were written in English were included in the research. A total of 7,087 publications were involved in this study. The retrieval strategy of our study is shown in Figure 1.

### Data Collection and Cleaning

First, we extracted original data from the WoSCC database. The number of publications, authors, citations, publication year, affiliations, H-index, references, countries/regions, journals, and keywords were included in the recorded information. Although inexact analysis could not fully be prevented because of different forms of cited journals, the same abbreviated name of different authors, and multiple versions of cited references, most raw data in our study were considered reliable. Data were analyzed by VOSviewer v.1.6.10.0 and CiteSpace 5.8.R3.

### Bibliometric Analysis

The number of articles and citations are important bibliometric indicators often used to indicate bibliographic materials. In this study, the number of publications (Np) was applied to evaluate production and the number of citations (Nc) was utilized to indicate impact because they are two main angles for assessing the research level (23). Co-citation is defined when both items are referenced by a third item. Keyword co-occurrence measures the keywords with the highest frequency in the same literature (24), and the analysis of co-occurrence keywords and co-cited references could explain research hotspots associated with PS. The H-index is used to assess researchers' academic contributions and predict scientific achievements in future (25). The H-index unifies productivity and impact by finding thresholds that connect Np and Nc. Thus, a researcher would have an H-index if he or she published H articles, and every article had been cited at least H times (26). In particular, the H-index could be employed to evaluate individual academic achievements and illustrate the publication of a journal, an affiliation, a country, or a region (27). To measure the impact and quality of journals, the impact factor (IF) obtained from Journal Citation Reports (JCR) has been widely regarded as a main indicator (28). Bibliometric maps were constructed to obtain a more comprehensive result according to co-occurrence and co-citation by using VOSviewer and CiteSpace in our study.

## Results

### Overview of Publications on PS

A total of 7,087 articles and reviews published from 2011 to 2021 were retrieved through the retrieval method. Among them, the total Nc was 121,855, the average Nc per item was 21.07, and all publications' H-index was 132.

### Annual Trend of Publication Quantity

**Figure 2:**
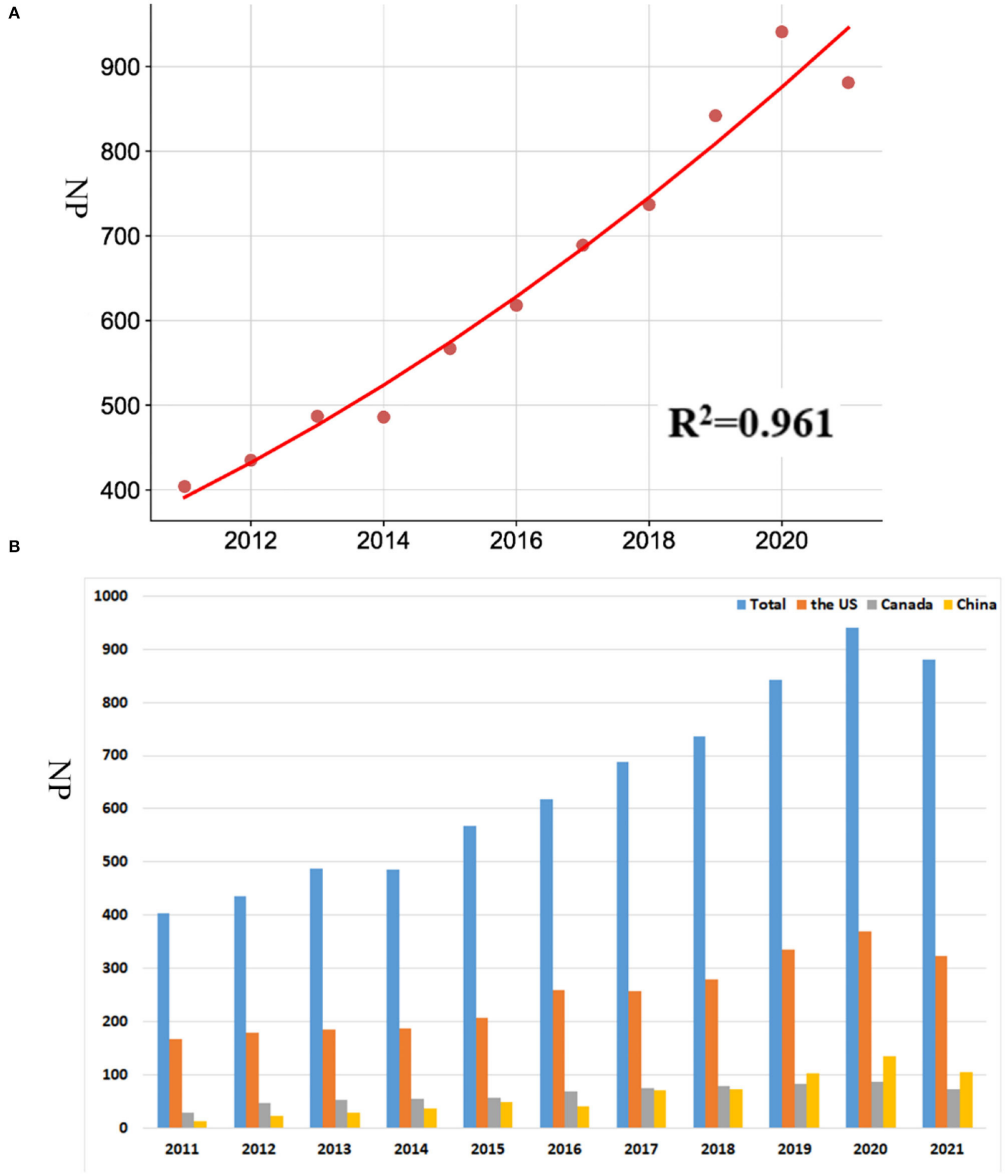
**(A)** Curve fitting of the total annual growth trend of publications. **(B)** Number of publications by year from 2011 to 2021.

The annual Np was associated with publication year, and the correlation coefficient (*R*^2^) was 0.961 (Figure 2A). The annual number of articles published increased from 404 in 2011 to 941 in 2020, and Np achieved the crest value in 2020. In the United States, China, and Canada, the annual Np generally maintains an increasing trend (Figure 2B). The polynomial fitting curve illustrated an annual trend of publication quantity (Figure 2A). This indicated that investigations on PS have rapidly developed with time.

### Contributions of Countries/Regions

**Figure 3:**
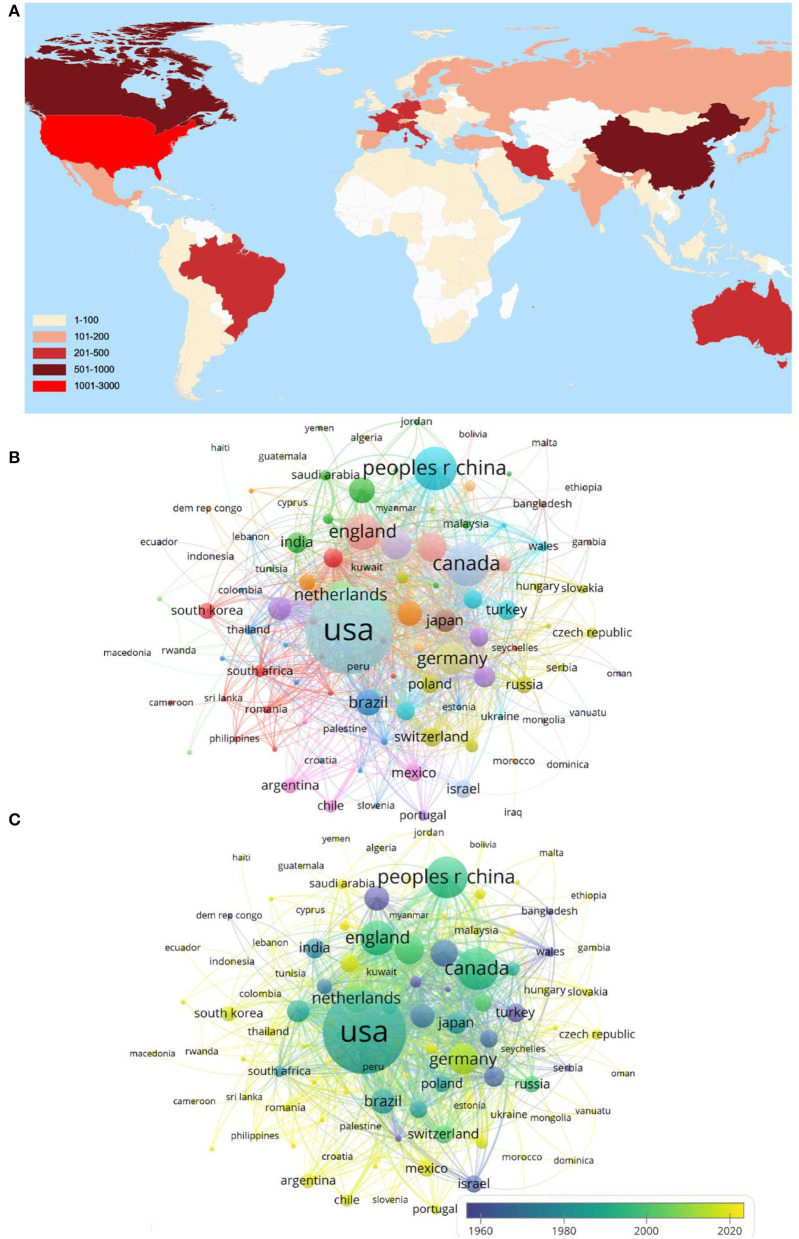
Mapping of countries of studies related to PS. **(A)** Geographic distribution of PS-related research articles from 2011 to 2021. **(B)** Network of 110 countries. **(C)** Visualization of countries according to the APY. Countries in yellow published articles later than those in blue.

The top 10 high-output countries/regions according to Np are listed in Table 1. The United States ranked first and published the most articles (2,751/38.82%). Canada (702/9.91%) ranked second, and China (674/9.5%) ranked third. The number of publications in the United States was more than triple the figure for Canada or China. Articles published by the United States were cited 63,711 times, accounting for 52.28% of the total Nc, followed by Canada (17,870) and England (13,464). Furthermore, the United States had the highest H-index (108), which was more than two times the figure obtained for China (41). The Np in England and Germany was slightly lower, but their H-index, Nc, and average citation per item were fairly higher than those of China. Figure 3A shows the number of articles published by different countries and regions. As shown in Figure 3B, the United States and Canada are central to this research field, and Figure 3C shows that the two countries conduct research in this field earlier than other countries.

**Table 1:** Top 10 productive countries/regions.

**Rank**	**Country**	**NP**	**NC**	**H-index**	**Average citation per item**
1	the US	2,751	63,711	108	25.42
2	Canada	702	17,870	68	27.49
3	China	674	7,875	41	12.54
4	England	463	13,464	58	30.19
5	Germany	371	9,086	51	25.53
6	Australia	334	7,257	41	22.35
7	Netherlands	299	8,960	53	31.52
8	Italy	290	6,070	39	21.76
9	Iran	239	2,024	23	9.59
10	Brazil	237	3,616	30	15.68

### Analysis of Affiliations

**Figure 4:**
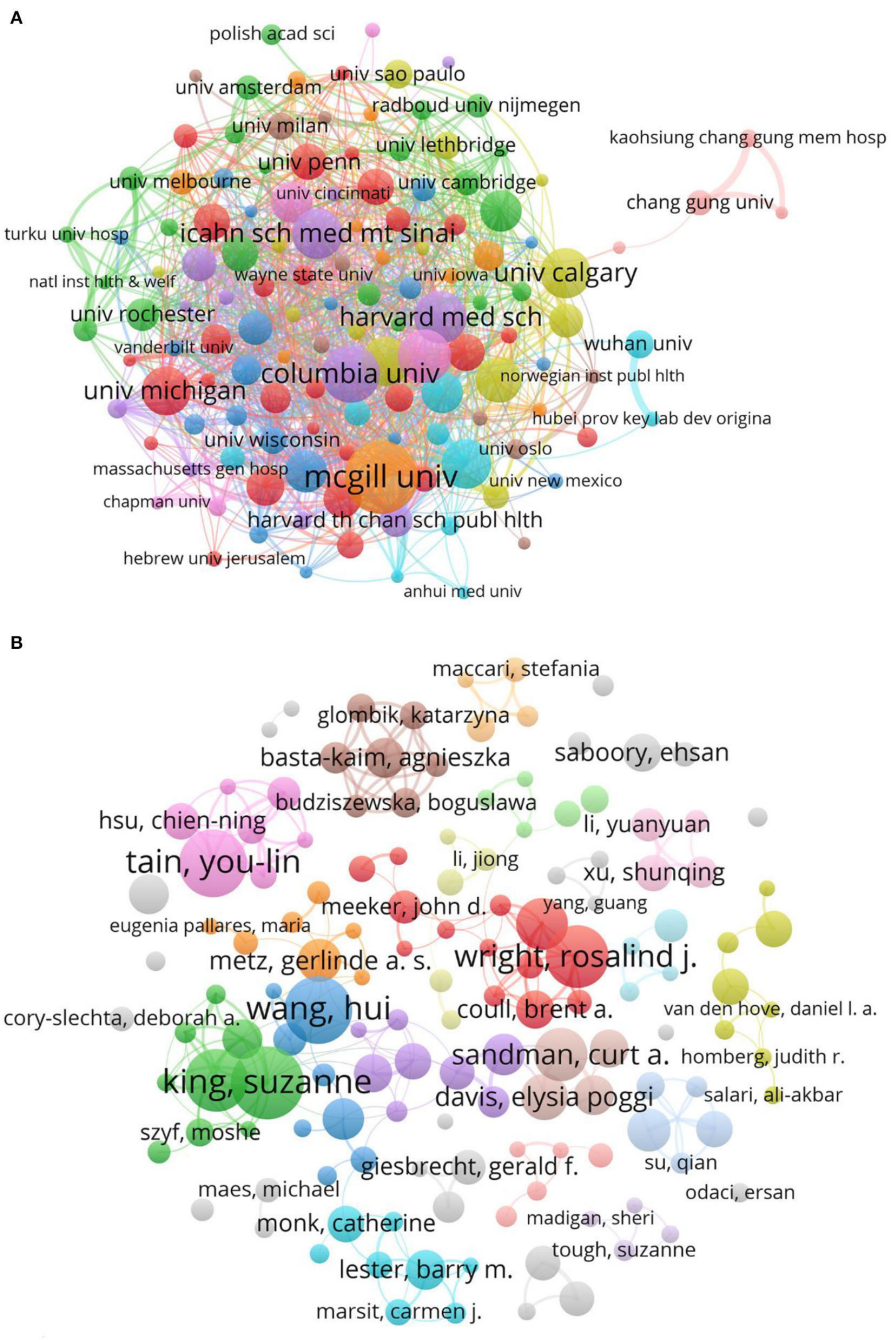
Network of affiliations and authors related to PS. **(A)** Network of affiliations. Of the 5,601 affiliations, 131 affiliations had at least 25 documents. **(B)** Network of authors. Of the 28,795 authors, 140 authors had at least 10 documents.

The top 10 affiliations with the largest number of publications related to PS are shown in Table 2. The University of California system ranked first in terms of Np, followed by Harvard University and the University of London. The University of California system also had the highest Nc (12,376), H-index (59). Most affiliations were from the United States, except the University of London in England, McGill University, and the University of Toronto in Canada. Figure 4A shows the co-occurrence of different affiliations.

**Table 2:** Top 10 productive affiliations.

**Rank**	**Affiliation**	**NP**	**NC**	**Country**	**H-index**	**Average citation per item**
1	UNIVERSITY OF CALIFORNIA SYSTEM	375	12,376	the US	59	34.39
2	HARVARD UNIVERSITY	213	4,555	the US	38	22.01
3	UNIVERSITY OF LONDON	156	5,599	England	39	36.61
4	MCGILL UNIVERSITY	140	4,549	Canada	38	34.93
5	PENNSYLVANIA COMMONWEALTH SYSTEM OF HIGHER EDUCATION PCSHE	127	2,499	the US	26	20.16
6	ICAHN SCHOOL OF MEDICINE AT MOUNT SINAI	116	2,894	the US	30	25.77
7	COLUMBIA UNIVERSITY	113	3,234	the US	33	29.35
8	UNIVERSITY OF NORTH CAROLINA	110	2,080	the US	25	19.13
9	UNIVERSITY OF TORONTO	109	3,501	Canada	29	32.83
10	HARVARD T H CHAN SCHOOL OF PUBLIC HEALTH	108	2,415	the US	31	22.96

### Performance of Authors

The top 10 authors with the most publications published 391 articles, which accounted for 5.52% of the total articles (Table 3). Wang H from the Wuhan University ranked first in the field of PS investigation, followed by King S from McGill University in Canada and Tain YL from Chang Gung University in Taiwan, China. Sandman CA had an high Nc. A majority of the top 10 authors were from the United States and Canada. Figure 4B shows the co-occurrence of different authors.

**Table 3:** Top 10 authors with the most publications.

**Rank**	**Author**	**NP**	**NC**	**Country**	**Affiliation**	**H-index**	**Average citation per item**
1	Wang H	52	496	China	WuHan Univ	13	11.6
2	King S	49	1,365	Canada	McGill Univ	22	32.59
3	Tain YL	43	690	China Taiwan	Chang Gung Univ	19	20.88
4	Wright RJ	43	810	the US	Icahn Sch Med Mt Sinai	19	20.14
5	Laplante DP	41	995	Canada	Mcgill Univ	19	28.1
6	Li H	35	502	China	Xi An Jiao Tong Univ	12	15.29
7	Wright RO	34	471	the US	Icahn Sch Med Mt Sinai	13	15.03
8	Sandman CA	33	2,066	the US	Univ Calif Irvine	21	66.3
9	Li J	31	329	Denmark	Aarhus Univ	11	11.06
10	Davis EP	30	1,804	the US	Univ Calif Irvine	17	63.83

### Analysis of Journals

The top 10 journals with the most publications are shown in Table 4. PLOS One (174 publications, IF: 3.24) published the most articles related to PS, followed by Psychoneuroendocrinology (122 publications, IF: 4.905) and Scientific Reports (88 publications, IF: 4.38). About 12.98% of the retrieved publications were in the 10 journals (920/12.98%). In the 10 journals, except International Journal Of Developmental Neuroscience (IF: 2.457), the rest were journals with a high IF (defined as >3.000), and Psychoneuroendocrinology (IF = 4.905) had a higher average citation per item (34.59).

**Table 4:** Top 10 most active journals.

**Rank**	**Journal**	**NP**	**NC**	**IF (2020)**	**H-index**	**Average citation per item**
1	PLOS ONE	174	4,271	3.24	37	24.68
2	PSYCHONEUROENDOCRINOLOGY	122	4,096	4.905	37	34.59
3	SCIENTIFIC REPORTS	88	1,269	4.38	22	14.56
4	BEHAVIORAL BRAIN RESEARCH	86	1,528	3.332	23	18.27
5	JOURNAL OF AFFECTIVE DISORDERS	81	2,395	4.839	24	30.27
6	DEVELOPMENTAL PSYCHOBIOLOGY	80	1,222	3.038	22	15.65
7	INTERNATIONAL JOURNAL OF MOLECULAR SCIENCES	77	1,048	5.924	17	14.27
8	INTERNATIONAL JOURNAL OF ENVIRONMENTAL RESEARCH AND PUBLIC HEALTH	73	403	3.39	11	5.55
9	BMC PREGNANCY AND CHILDBIRTH	70	958	3.007	17	13.81
10	INTERNATIONAL JOURNAL OF DEVELOPMENTAL NEUROSCIENCE	69	836	2.457	18	12.41

### Analysis of Article Global Citations (GCS)

**Figure 5:**
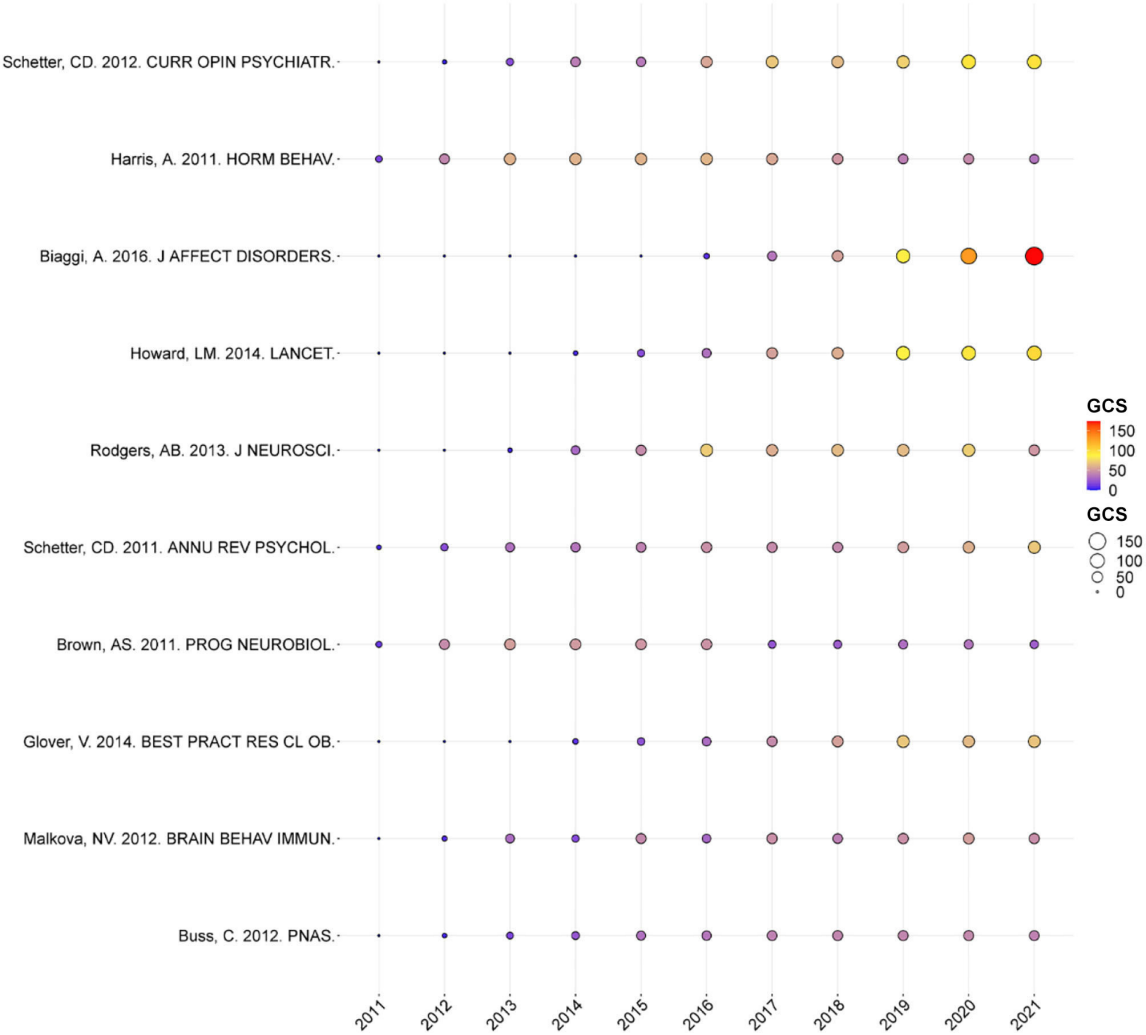
Yearly number of global citations of articles with high global citations (GCS). The GCS of each article is shown by the size and colors of the circle.

The annual number of GCS for the top 10 articles is shown in Figure 5. The article published in CURR OPIN PSYCHIATR and written by Schetter CD in 2012 ranked first, with a GCS of 551. In this article, the authors pointed out that risk factors, such as stress, anxiety, and depression, had adverse effects on children and mothers during pregnancy. Anxiety in pregnancy, which was associated with shorter gestation, had adverse effects on child outcomes such as fetal neurodevelopment. Anxiety was especially potent during pregnancy. During pregnancy, exposure to racism, chronic strain, and depressive symptoms in mothers were related to lower birth weight in infants. These risk factors which could be distinguished and with pathways associated with distinct birth outcomes need further investigation (29). Harris et al. summarized that an adverse fetal environment was related to an increased risk of psychological, cardiovascular, neuroendocrine, and metabolic disorders in adulthood (30). Biaggi et al.'s study showed the complicated etiology of antepartum anxiety and depression. Knowing specific risk factors might help to create screening tools for high-risk women, and the use of screening tools to identify women at risk of anxiety and depression during pregnancy should be common practice (31). Howard et al. pointed out that, during pregnancy, mental disorder was one of the most common diseases that could be harmful to the family, mother, and child, and they summarized the evidence on interventions, risk factors, epidemiology, and identification for nonpsychotic mental disorders (32). Schetter pointed out that depression and chronic stress were the key risk factors in the etiology of low birth weight and anxiety in pregnancy in the etiology of preterm birth. The effect of PS on neural development was emphasized, and the resilience resources of pregnant women were conceptualized (33). Brown discussed the role of environmental risk factors during perinatal and fetal life (34). Glover showed that, during pregnancy, the mother's emotional state could influence her child for a long time, especially in the development of the nervous system. The mechanisms of fetal programming were just beginning to be revealed, and the correlation between psychometric indicators and biological indicators was often limited. Therefore, it was necessary to determine the most effective interventions during pregnancy in improving child outcomes (35). Malkova et al.'s study provided a new insight that, during pregnancy, environmental risk factors induced autistic-like behaviors in offspring (36). Research of Buss et al. emphasized the importance of the intrauterine environment and suggested that the early stage of life might be accompanied with the origin of neuropsychiatric disorders (37). In general, these publications were different in the research of PS and had far-reaching significance, and these publications raised the number of subsequent publications in the field.

### Co-Cited Reference Analysis

**Figure 6:**
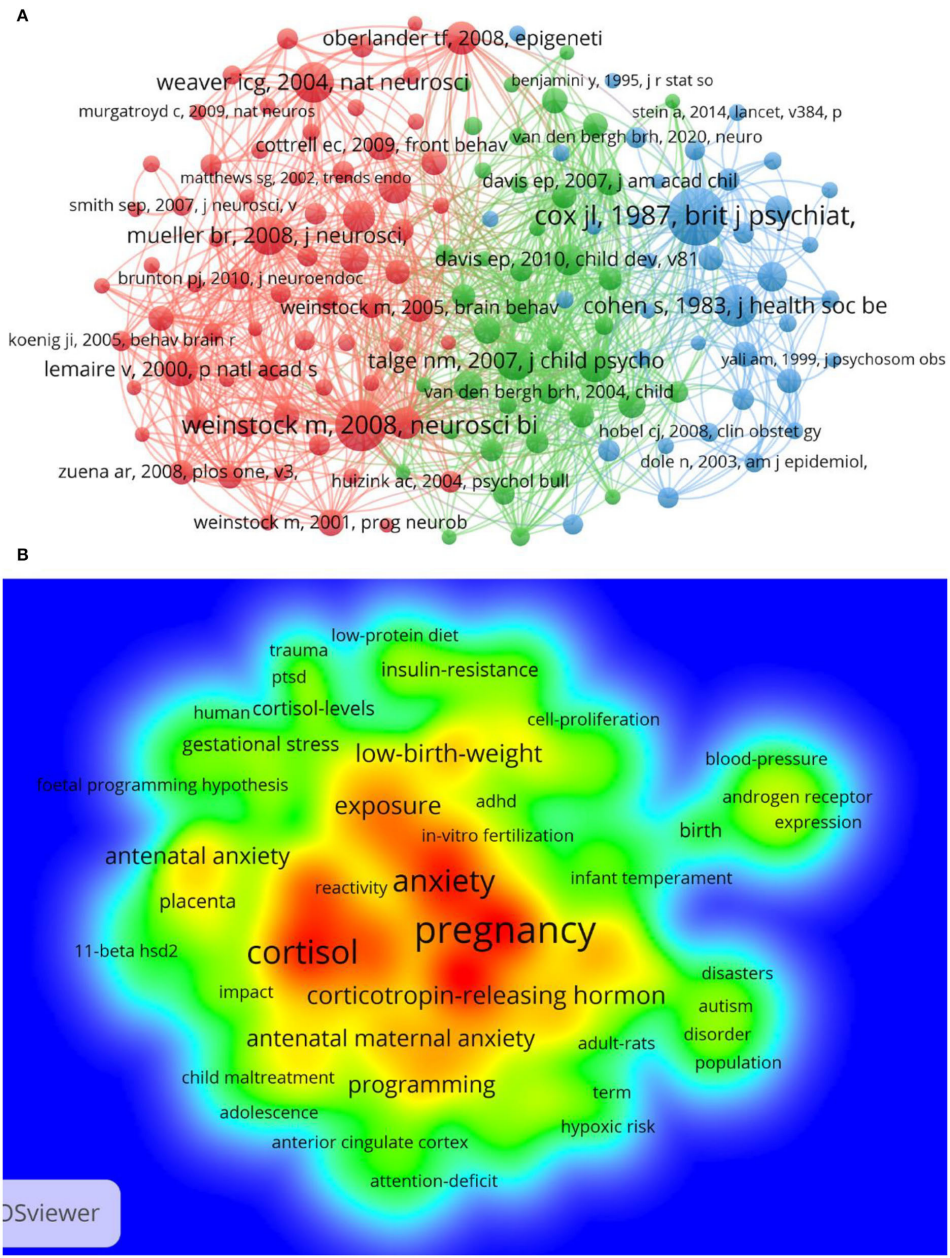
Co-cited reference network of PS-related research. **(A)** Network of co-cited references. Of the 2,71,109 references, 149 references were cited at least 76 times. **(B)** Density visualization of keywords of cluster 2 in the network of co-cited references. Each keyword in density visualization has a color indicating its density. Red indicates high frequency, and yellow indicates low frequency.

The co-citation network emphasizes research topics closely related to specific fields, which is different from global citation analysis (38). The minimum number of citations per reference was 76 due to a large number of cited references. A total of 271,109 references were cited by the retrieved publications, and we selected 149 references for co-citation analysis (Figure 6A). The line between two nodes indicated that both nodes were cited in an article, and the shorter line denoted that the relationship between the two articles was closer. The size of the node represented the total link strength and the total number of common references to the document. In addition, references were divided into different clusters using different color nodes. There were 69 references in cluster 1 (in red), which mainly elaborated the effect of PS on offspring and pathogenesis. Cluster 2 (in green) included 41 references mainly focused on clinical research, which showed that PS could cause many adverse effects on offspring. Cluster 3 (in blue) included 39 references centered on effects of anxiety, depression, and stress exposure during pregnancy on adverse maternal and infant outcomes. Research on the HPA axis in PS and interventions might be helpful. Research into different clusters has a certain relationship, but the relationship is not close. For further studies on the co-citation of clinical research and underlying mechanism references, 28 references in cluster 2 (13 references not found in WoSCC were excluded) were analyzed through density visualization. Density visualizations usually highlight important study fields and present the whole structure of the research (39). Clinical research and underlying mechanisms of PS were the themes of cluster 2 co-cited references (Figure 6B). For instance, “depression,” “anxiety,” “fetal programming,” and “cortisol” were widely cited. References about clinical research in PS such as “antenatal maternal anxiety,” “children,” “birth,” “mothers,” and “infant temperament” were mainly cited. Moreover, the keyword “cortisol” was in the middle of the map, and it appeared more than once, suggesting that the topic was closely associated with PS and should be further explored.

### Research Hotspots Analysis

**Figure 7:**
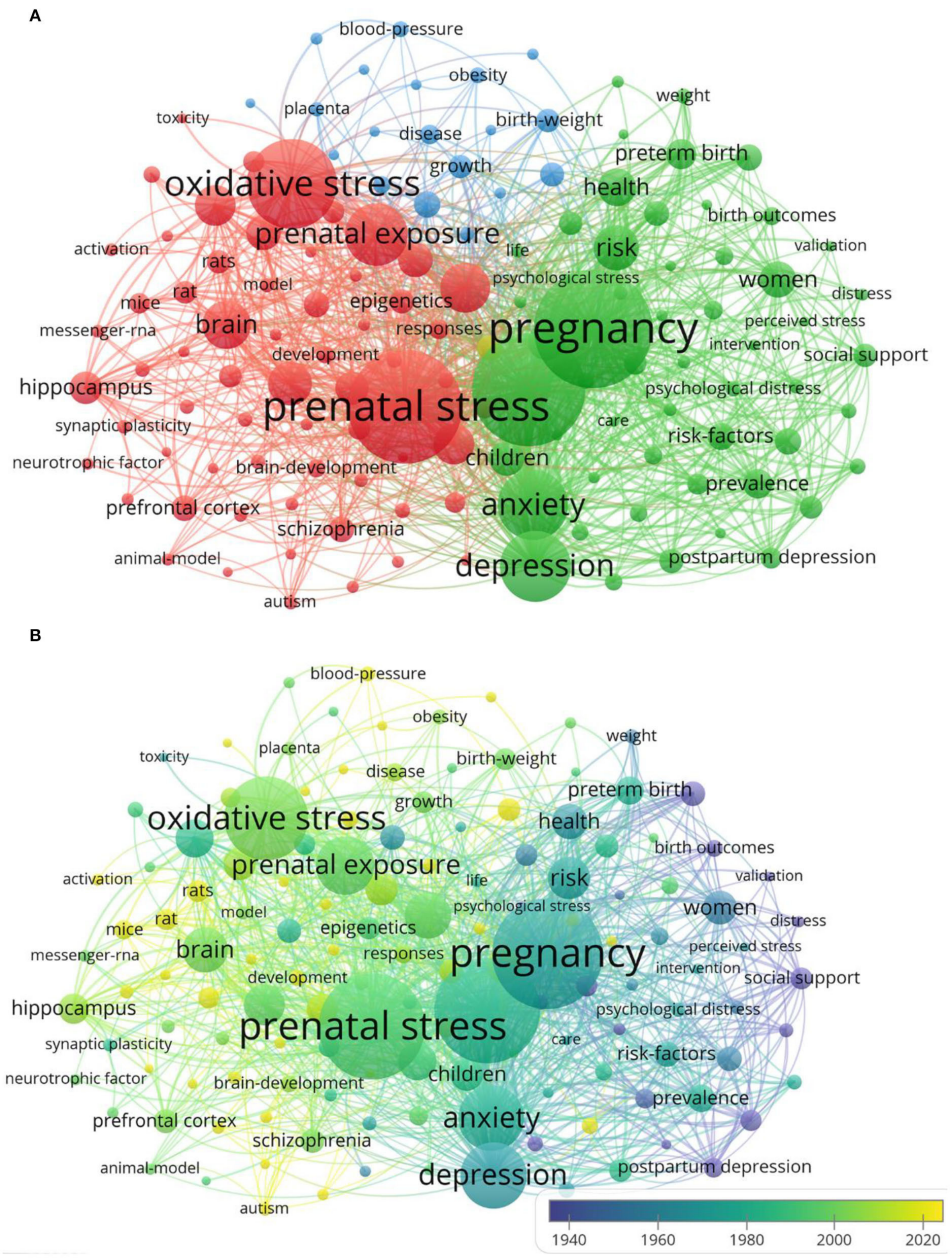
PS keyword network. **(A)** The 137 keywords that appear more than 80 times are divided into four categories according to different colors: the first category: red, the second category: green, the third category: blue, and the fourth category: yellow. The size of the node indicates the frequency of occurrence. **(B)** Visualization of the keywords according to APY. The yellow keywords appear later than the blue keywords.

**Figure 8:**
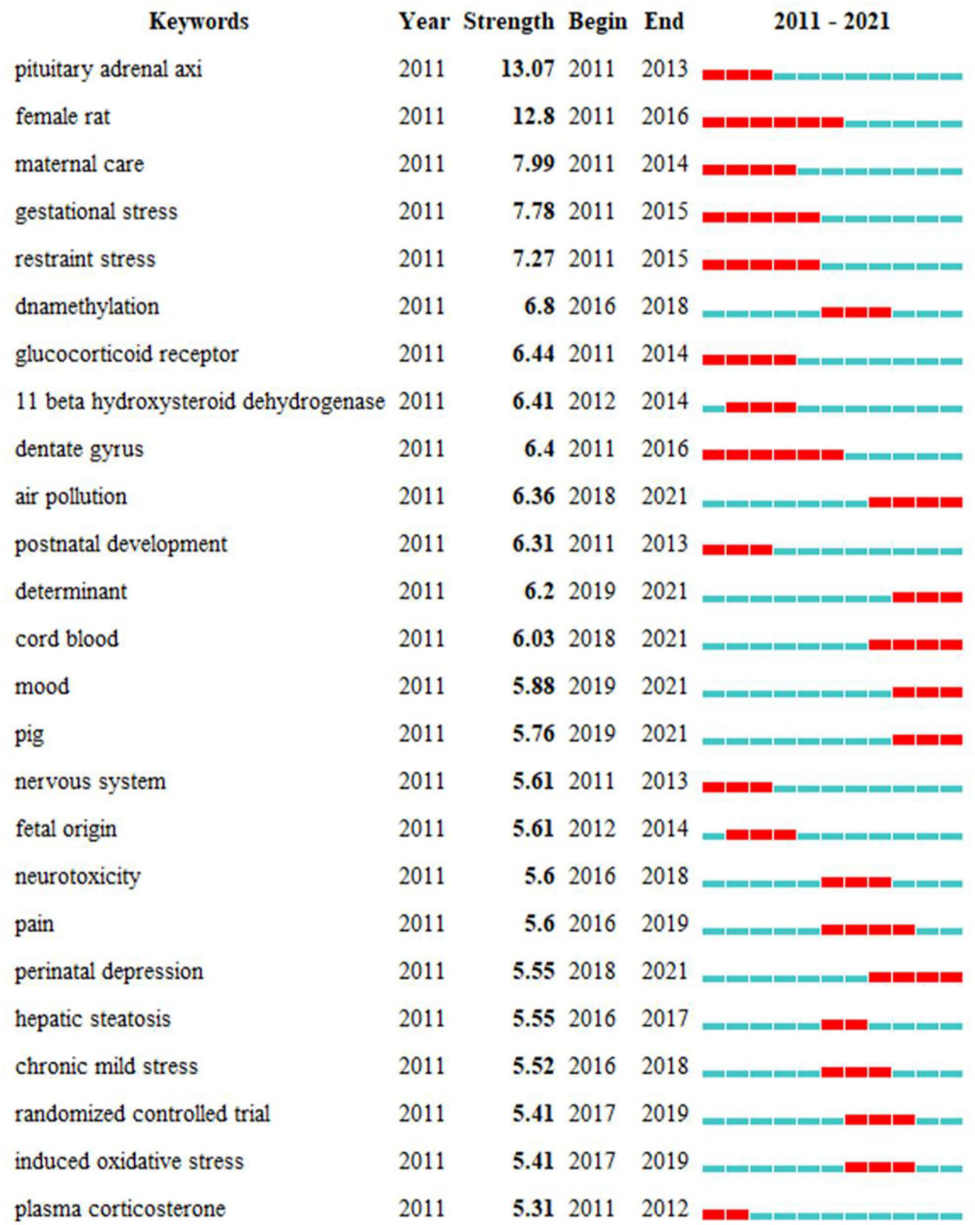
Top 25 representative burst keywords.

Figure 7A shows that cluster 1 mainly focused on basic research on PS, cluster 2 was mainly about clinical research on PS and explored the effects of PS on newborn growth and development, and cluster 3 investigated prenatal risk factors and adverse consequences caused by PS. The most frequent occurrences of keywords were “prenatal stress,” “pregnancy,” “stress,” “oxidative stress,” and “depression,” indicating that studies associated with PS mainly concentrated on clinical studies. In Figure 7B, based on the average publication year (APY), we divided the colors of all keywords using VOSviewer. The keywords with a high frequency in recent years were as follows: “salivary cortisol” (cluster 2, APY: 2016, 40), “*in utero*” (cluster 3, APY: 2016, 67), “autism” (cluster 3, APY: 2017, 13), and “autism spectrum disorder” (cluster 1, APY: 2018, 55). In addition, “cortisol,” “oxidative stress,” and “DNA methylation” were the main topics in this field. The applications of epigenetics to studies on PS and low birth weight of infants were relatively latest compared with those illustrated in Figures 7A,B. In addition to the search terms, the keywords extracted from abstracts and titles of 7,087 publications (Figure 8) were analyzed using CiteSpace. As shown in Figure 8, among the top 25 representative burst keywords, “air pollution,” “cord blood,” “determinate,” “mood,” and “pig,” were the most novel. “Female rat,” “pig,” and “restraint stress” were the terms of basic research, and “randomize controlled trial,” “cord blood,” and “mood” were the terms of clinical research, which indicates that the retrieval of this study included clinical and basic research studies. Furthermore, the latest research hotspots in 2021 are shown in Table 5.

**Table 5:** Latest research hotspot.

**Emerging in 2021**	**Owning the largest burstness in 2021**
1st year, antenatal education, anxiety like behavior, anxiety symptom, autophagy, brain barrier impairment, breast milk, complementary, conception, coping style, creb, developmental delay, developmental plasticity, discrimination, disturbance, endocrine, endothelial function, epigenetic mechanism, externalizing problem, ethnic disparity, glucose homeostasis, heart susceptibility, nrf2, phosphorylation, postpartum depressive symptom, prediction, profile, progesterone, race, reproduction, time	Air pollution, determinant, cord blood, mood, pig, perinatal depression

### Analysis of Research on PS Caused by COVID-19

**Figure 9:**
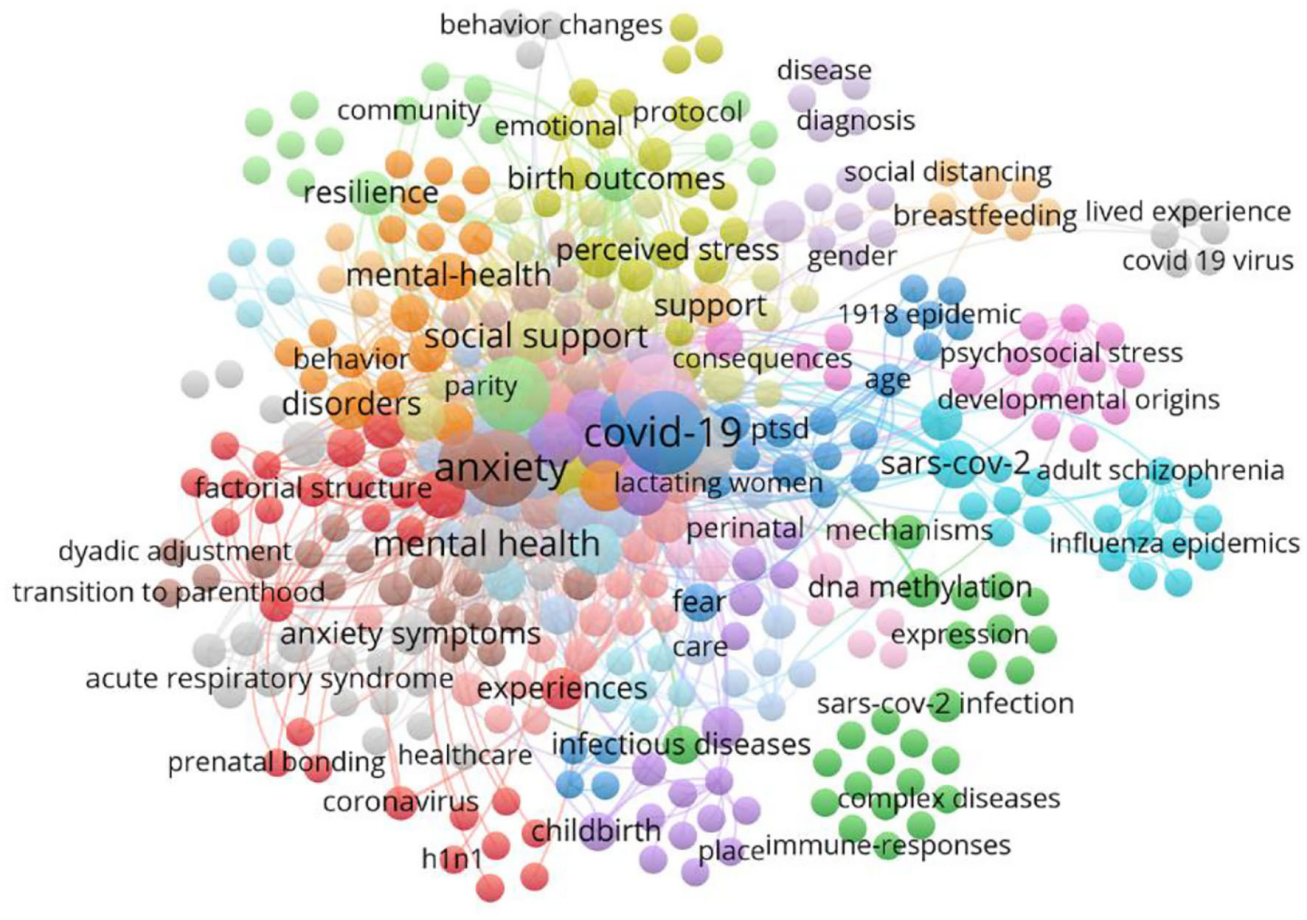
Network of keywords of PS caused by COVID-19, and 367 keywords are divided into 22 categories according to different colors. The size of the node indicates the frequency of occurrence.

The COVID-19 pandemic not only caused social panic but also had a negative impact on the physical and mental health of pregnant women and newborns. Therefore, research publications on PS related to COVID-19 were analyzed. The Np associated with PS in 2020 increased sharply during the COVID-19 pandemic (Figure 2A). To further understand the hotspots and trends associated with PS induced by COVID-19, we screened and analyzed 78 publications about PS associated with COVID-19 among the 7,087 publications we retrieved. Except for COVID-19, pregnancy, anxiety, stress, depression, and mental health occurred most frequently (Figure 9). Furthermore, Figure 9 presents the pathogenesis of PS induced by COVID-19. COVID-19 caused panic among the public. During this period, pregnant women and babies were negatively affected to varying degrees, influencing the brain development, weight, emotion, and psychology of infants and young children. The degree of its impact has reached the molecular level, such as in genes. Therefore, during the COVID-19 pandemic, women should take corresponding protective measures to reduce or even avoid the interference of adverse factors during pregnancy. Medical institutions should also strengthen the psychological health care of pregnant women in this period.

### Bibliographic Coupling Analysis

**Figure 10:**
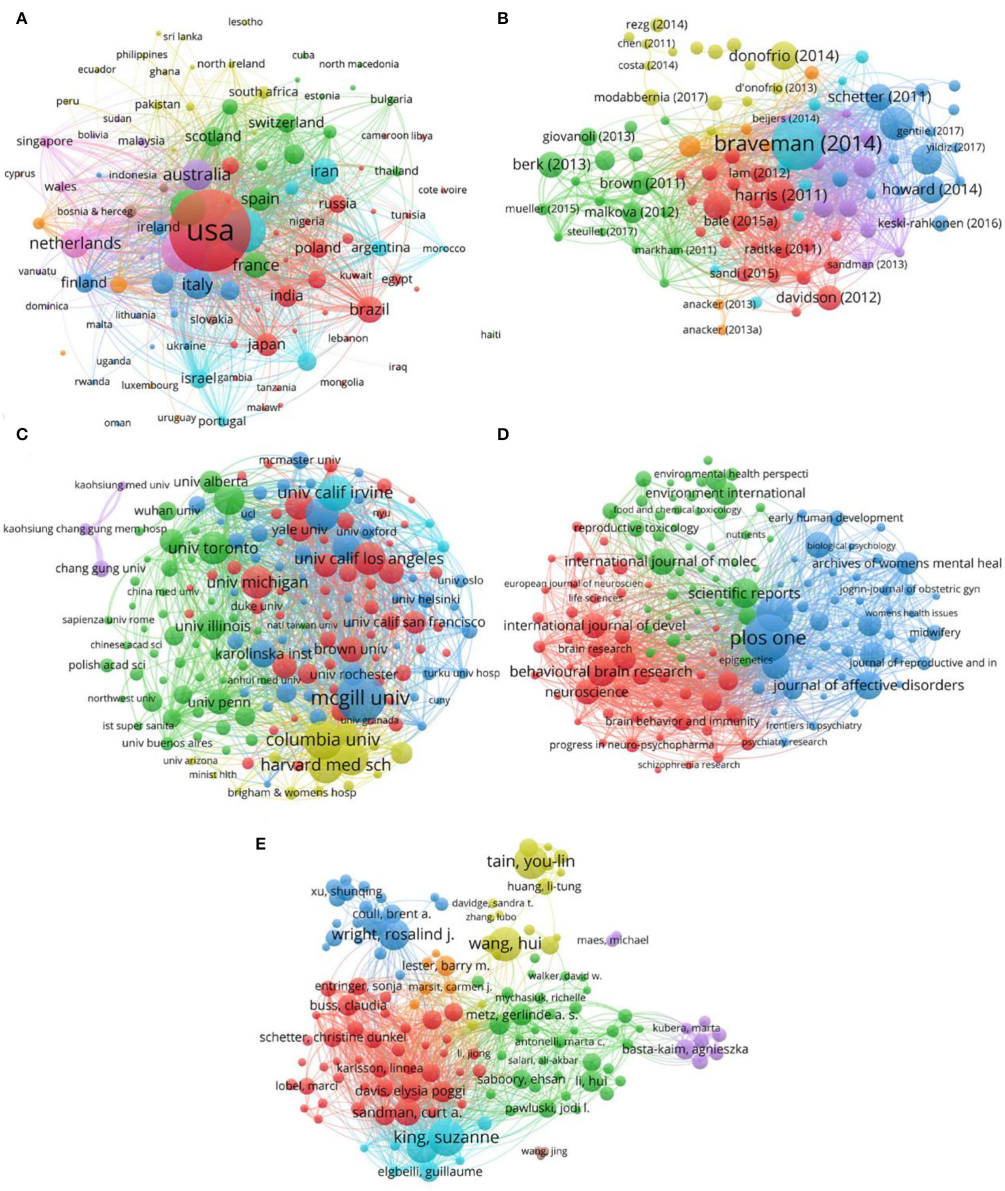
Bibliographic coupling analysis of PS-related research. **(A)** Network of bibliographic coupling countries. **(B)** Network of bibliographic coupling documents. **(C)** Network of bibliographic coupling affiliations. **(D)** Network of bibliographic coupling journals. **(E)** Network of bibliographic coupling authors.

Bibliographic coupling refers to two articles quoting one or more references from the same article. Usually, the number of citation coupling can be used to quantitatively measure the static relationship between the two articles. The more the number of citation coupling, the stronger the correlation between the two articles. The strength of the coupling depends on the number of common references (citations). Bibliographic coupling can be divided into article coupling, discipline coupling, author coupling, and journal coupling. In addition, there are country coupling and language coupling. Bibliographic coupling is applied to the fields of information science, bibliometrics, science, etc. The analysis of bibliographic coupling is shown in Figure 10. The top 10 countries were the United States (69,885 times), Canada (19,296 times), England (13,651 times), Germany (9,441 times), the Netherlands (9,385 times), China (8,450 times), Australia (7,457 times), Italy (6,309 times), France (4,741 times), and Switzerland (4,367 times). The top 10 documents were Braveman (2014, 904 times), Schetter (2012, 568 times), Harris (2011, 531 times), Biaggi (2016, 513 times), Donofrio (2014, 483 times), Howard (2014, 465 times), Rodgers (2013, 463 times), Schetter (2011, 460 times), Berk (2013, 441 times), and Davidson (2012, 424 times). The top 10 affiliations were McGill University (4,855 times), the University of California Irvine (4,607 times), the University of Pennsylvania (3,857 times), King's College London (3,502 times), the University of California, Los Angeles (3,460 times), Columbia University (3,276 times), the University of Toronto (2,891 times), the University of California San Francisco (2,874 times), the University of Calgary (2,555 times), and the University of British Columbia (2,492 times). The top 10 journals were PLOS One (4,294 times), Psychoneuroendocrinology (4,220 times), Neuroscience (2,769 times), Journal of Affective Disorders (2,452 times), Neuroscience & Biobehavioral Reviews (2,198 times), Proceedings of the National Academy of Sciences of the United States (2,198 times), Biological Psychiatry (2,038 times), Translational Psychiatry (1,838 times), Hormones and Behavior (1,743 times), and Behavioral Brain Research (1,571 times). The top 10 authors were Bale, Tracy L. (3,138 times), Sandman, Curt A. (2,181 times), Glover, Vivette. (1,939 times), Davis, Elysia Poggi. (1,868 times), Schetter, Christine Dunkel. (1,796 times), Buss, Claudia. (1,644 times), Entringer, Sonja. (1,526 times), King, Suzanne. (1,483 times), Glynn, Laura M. (1,304 times), and Wadhwa, Pathik D. (1,253 times).

## Discussion

In our study, a bibliometric analysis was conducted on the basis of the WoSCC database using VOSviewer to explore the hotspots and developmental trends of investigations on PS. A total of 7,087 reviews and original articles published from 2011 to 2021 were retrieved. On the whole, the number of annual publications showed a gradual upward trend, and the largest number of documents was published in 2020 based on the polynomial fitting curve, which showed that the heat and depth of academic studies on PS are increasing with each passing year. The United States ranked first among the top countries/regions in the Np, which indicated that it was a high-yielding country in this field. In research on PS, seven American affiliations and four American authors belonged to the top 10 affiliations and authors, suggesting in this field, the United States possessed the best affiliations and professional researchers in the world, and the United States has a high Nc and H-index, indicating that the United States has developed rapidly in this field in the past 11 years. Nevertheless, countries other than Iran had a relatively higher average citation per item than China because they have conducted more in-depth research studies. Thus, researchers and affiliations in China should focus on enhancing the quality of studies on PS. Similarly, the quantity and quality of publications in Iran have some contradictions. Furthermore, of the top 10 productive journals, nine journals had high IF, which indicated that publishing PS research in high-quality journals is not difficult. Among the top 10 articles with high GCSs, one article was published in The Lancet and one was published in PNAS. This finding suggested that The Lancet and PNAS had published some potential research breakthroughs on PS. It also reminds researchers interested in this field to consider publishing articles in these journals.

In recent years, researchers have focused on the complex function of epigenetics and mechanistic research. In-depth studies have revealed signal pathways involved in various mechanisms. Rodgers et al.'s study demonstrated that, throughout adolescence or adulthood, male exposure to stress reprogrammed paternal germ cells and led to the transmission of HPA stress axis dysregulated phenotypes in offspring. Whether this reduction in stress responsiveness was harmful or beneficial to offspring may depend on their birth environment and genetic background factors. However, it provides a novel and important mechanism for the increased risk of neuropsychiatric diseases that mild stress could produce long-term changes in male germ cells (40).

The network of keywords indicates that cortisol plays a major role in the pathogenesis of PS. As a glucocorticoid hormone and major regulatory factor produced by the adrenal glands, cortisol increases when a person is stressed. The empirical literature has used the perceived stress scale as a psychological tool by which cortisol levels were commonly used as an index to measure maternal stress during pregnancy. In recent years, in addition to cortisol levels in blood, urine, and saliva, the hair cortisol concentration (HCC) has also attracted extensive attention as a biomarker of stress (41). Cortisol levels in saliva, blood, and urine are suitable for representing acute stress, whereas the HCC can serve as a biomarker of chronic stress. The HCC is associated with fetal gestational age (42). Moreover, the application of molecular biology and epigenetics in PS has become a hotspot over the last 5 years, as shown in Figures 7A,B. Cao Lei et al. pointed out that uterus exposure to environmental stress may lead to long-term epigenomic changes that have adaptation and developmental consequences in human and animal offspring. One of the most well-defined and widely studied mechanisms on the long-term effects of uterostress exposure is epigenetics, specifically DNA methylation. To support such an idea, evidence from prenatal studies on animals and humans that PS may result in persistent, widespread, and functionally ordered features of DNA methylation has been presented. In turn, DNA methylation may mediate exposed phenotypic associations. The advantages of using stressors in quasi-random assignment experiments have also been emphasized. Furthermore, there are future challenges that still need to be addressed in this area. Genes may change the transcription or alternative splicing of target genes in tissues through epigenetic changes (including non-coding RNA and DNA methylation, downstream functional changes, and histone posttranslational modification) and retain the memory of early metabolic stress (43). The outbreak of COVID-19 in 2019 could adversely affect pregnant women and fetuses. Figure 9 shows that “fear,” “social distancing,” and “anxiety” “disorders” frequently occurred, showing that COVID-19 exerts a negative impact on pregnant women and newborns. COVID-19 is a new stressor that influences individuals preparing for childbirth in a phenomenon called COVID-19-specific prenatal distress. This disease can exacerbate stress during pregnancy and is associated with increased anxiety during pregnancy (44). The key to formulating strategies that support pregnant women during the COVID-19 pandemic is to identify potential risks and protective factors associated with general antenatal distress and COVID-19-specific prenatal distress. Emotional social support and interpersonal characteristics such as relationship satisfaction or a better relationship with mothers during pregnancy are related to decreased pregnancy-specific pain. However, during this pandemic, isolation measures limit access to resources such as prenatal care or caregiver support. Thus, tangible assistance such as financial support or assistance in household chores or instrumental support may become particularly important. Pain tolerance refers to a person's ability to manage and tolerate emotional pain. Psychological resilience refers to a person's ability to thrive in adversity or recover from challenges or setbacks.

With keyword burst analysis, we extracted a total of 170 keywords, and the top 25 keywords are shown in Figure 8. We found that the “pituitary–adrenal axis” and “female rat” had the highest burst strength. We also observed that “determinant,” “cord blood,” “mood,” “air pollution,” “perinatal depression,” and “pig” were the latest keywords that appeared in the last 4 years (Figure 8). Evidence of the contribution of prenatal household air pollutant (HAP) exposure to elevated oxidative stress in children is mounting. Increased prenatal HAP exposure could reduce CBMC mtDNAcn, indicating cumulative prenatal oxidative stress injury (45). A significant correlation exists between prenatal exposure to air pollutants and the level of SOD2 promoter methylation in umbilical cord blood, and the level of SOD2 promoter methylation in maternal peripheral blood may be the main influencing factor. This association may explain the mechanisms of oxidative stress and adverse health outcomes in children caused by prenatal air pollution (46). Liu et al. first reported the effects of prenatal exposure to polycyclic aromatic hydrocarbons (PAHs) and maternal stress on infant reactivity and regulation (47). They found that prenatal exposure to PAHs and maternal perceived stress jointly reduce the social ability of infants at 4 or 12 months after birth. Additionally, prenatal exposure to PAHs reduces selenium levels at 4 months, indicating increased behavioral problems at 12 months. Individual differences in reactivity and regulation could be reliably measured early in life, which can help identify children who may be at increased risk of psychosis owing to PAHs exposure. Previous studies have shown that increased prenatal ambient air pollution exposure is related to decreased umbilical cord blood (45, 48). The measurement of biomarkers in cord blood may provide a complete source of molecular information for fetal exposure during pregnancy. Keshavarzi et al. studied the relationship between maternal anxiety, maternal serum, and fetal cord blood cortisol, revealing that high maternal anxiety increases fetal cord blood cortisol and may regulate fetal growth (49). These studies show that cord blood plays an increasingly important role in PS. Moreover, the environmental quality needs to be improved and psychosocial stress should be reduced to improve the development outcomes of children, especially those from disadvantaged communities wherein environmental exposure is too high.

To a certain extent, we can understand the hotspots, mainstream research, and development trends in this field through the visualization of publications and bibliometric analysis. This study can better promote the understanding of important nodes in the development trend of PS research by setting GCS as an index. However, our research has several limitations. First, only articles and reviews published from the WoSCC were incorporated. Second, full texts of publications could not be analyzed with VOSviewer, so some information might be omitted. Last, this study excluded articles newly published in 2021; as such, our information might be outdated to some extent.

## Conclusion

Bibliometric analysis revealed that current research on PS is developing rapidly. The United States has contributed many outstanding scientific research achievements and breakthroughs in this field and ranked first among high-output countries. The University of California system has made novel progress and published most studies in this field. Epigenetics and maternal immunity activation in PS have become research hotspots. Moreover, children born during the COVID-19 pandemic need more attention and should be given more care.

## Data Availability Statement

The original contributions presented in the study are included in the article/supplementary material. Further inquiries can be directed to the corresponding authors.

## Author Contributions

YD, YM, SC, JX, and JC carried out this bibliometrics analysis and wrote the manuscript. GL, ZW, JZ, HM, and YR participated in experimental design and manuscript writing. HL and ZZ designed this study and organized the manuscript. All authors contributed to the article and approved the submitted version.

## Funding

This work was supported by the National Natural Science Foundation of China (Grant No. 82171516) and Department of Science and Technology of Shaanxi Province (2019ZDLSF04-02).

## Conflict of Interest

The authors declare that the research was conducted in the absence of any commercial or financial relationships that could be construed as a potential conflict of interest.

## Publisher's Note

All claims expressed in this article are solely those of the authors and do not necessarily represent those of their affiliated organizations, or those of the publisher, the editors and the reviewers. Any product that may be evaluated in this article, or claim that may be made by its manufacturer, is not guaranteed or endorsed by the publisher.

## References

[1] Guan SZ Fu YJ Zhao F Liu HY Chen XH Qi FQ. The mechanism of enriched environment repairing the learning and memory impairment in offspring of prenatal stress by regulating the expression of activity-regulated cytoskeletal-associated and insulin-like growth factor-2 in hippocampus. Environ Health Prev Med. (2021) 26:8. 10.1186/s12199-020-00929-7 33451279 PMC7811238

[2] Abramova O Ushakova V Zorkina Y Zubkov E Storozheva Z Morozova A. The behavior and postnatal development in infant and juvenile rats after ultrasound-induced chronic prenatal stress. Front Physiol. (2021) 12:659366. 10.3389/fphys.2021.659366 33935805 PMC8082110

[3] Roshan-Milani S Seyyedabadi B Saboory E Parsamanesh N Mehranfard N. Prenatal stress and increased susceptibility to anxiety-like behaviors: role of neuroinflammation and balance between GABAergic and glutamatergic transmission. Stress. (2021) 24:481–95. 10.1080/10253890.2021.1942828 34180763

[4] Lei L Wu XM Gu HW Ji MH Yang JJ. Differences in DNA methylation reprogramming underlie the sexual dimorphism of behavioral disorder caused by prenatal stress in rats. Front Neurosci. (2020) 14:573107. 10.3389/fnins.2020.573107 33192258 PMC7609908

[5] Scott H Phillips TJ Sze Y Alfieri A Rogers MF Volpato V. Maternal antioxidant treatment prevents the adverse effects of prenatal stress on the offspring's brain and behavior. Neurobiol Stress. (2020) 13:100281. 10.1016/j.ynstr.2020.100281 33344732 PMC7739187

[6] Luft C Haute GV Wearick-Silva LE Antunes KH da Costa MS de Oliveira JR. Prenatal stress and KCl-induced depolarization modulate cell death, hypothalamic-pituitary-adrenal axis genes, oxidative and inflammatory response in primary cortical neurons. Neurochem Int. (2021) 147:105053. 10.1016/j.neuint.2021.105053 33961947

[7] Schroeder R Sridharan P Nguyen L Loren A Williams NS Kettimuthu KP. Maternal P7C3-A20 treatment protects offspring from neuropsychiatric sequelae of prenatal stress. Antioxid Redox Signal. (2021) 35:511–30. 10.1089/ars.2020.8227 33501899 PMC8388250

[8] Rakers F Rupprecht S Dreiling M Bergmeier C Witte OW Schwab M. Transfer of maternal psychosocial stress to the fetus. Neurosci Biobehav Rev. (2020) 117:185–97. 10.1016/j.neubiorev.2017.02.019 28237726

[9] Arutjunyan AV Kerkeshko GO Milyutina YP Shcherbitskaia AD Zalozniaia IV. Prenatal stress in maternal hyperhomocysteinemia: impairments in the fetal nervous system development and placental function. Biochemistry. (2021) 86:716–28. 10.1134/S0006297921060092 34225594

[10] Anifantaki F Pervanidou P Lambrinoudaki I Panoulis K Vlahos N Eleftheriades M. Maternal prenatal stress, thyroid function and neurodevelopment of the offspring: a mini review of the literature. Front Neurosci. (2021) 15:692446. 10.3389/fnins.2021.692446 34566560 PMC8455916

[11] Grundwald NJ Benitez DP Brunton PJ. Sex-dependent effects of prenatal stress on social memory in rats: a role for differential expression of central Vasopressin-1a receptors. J Neuroendocrinol. (2016) 28:1–14. 10.1111/jne.12343 26613552 PMC4950027

[12] Dowell J Elser BA Schroeder RE Stevens HE. Cellular stress mechanisms of prenatal maternal stress: heat shock factors and oxidative stress. Neurosci Lett. (2019) 709:134368. 10.1016/j.neulet.2019.134368 31299286 PMC7053403

[13] Walker DJ Zimmer C Larriva M Healy SD Spencer KA. Early-life adversity programs long-term cytokine and microglia expression within the HPA axis in female Japanese quail. J Exp Biol. (2019) 222:1–11. 10.1242/jeb.187039 30814294

[14] Barrett ES Swan SH. Stress and androgen activity during fetal development. Endocrinology. (2015) 156:3435–41. 10.1210/en.2015-1335 26241065 PMC4588834

[15] Merlot E Couret D Otten W. Prenatal stress, fetal imprinting and immunity. Brain Behav Immun. (2008) 22:42–51. 10.1016/j.bbi.2007.05.007 17716859

[16] Gao Y Wang F Song Y Liu H. The status of and trends in the pharmacology of berberine: a bibliometric review 1985-2018. Chin Med. (2020) 15:7. 10.1186/s13020-020-0288-z 31988653 PMC6971869

[17] Chen P Lin X Chen B Zheng K Lin C Yu B. The global state of research and trends in osteomyelitis from 2010 to 2019: a 10-year bibliometric analysis. Ann Palliat Med. (2021) 10:3726–38. 10.21037/apm-20-1978 33832287

[18] Xing Y Ma Z Su W Wang Q Wang X Zhang H. Analysis of research status of CO2 conversion technology based on bibliometrics. Catalysts. (2020) 10:370. 10.3390/catal10040370

[19] Ding Y Chowdhury GG Foo S. Bibliometric cartography of information retrieval research by using co-word analysis. Inf Process Manag. (2001) 37:817–42. 10.1016/S0306-4573(00)00051-0

[20] Kokol P Blazun Vosner H Zavrsnik J. Application of bibliometrics in medicine: a historical bibliometrics analysis. Health Inf Libr J. (2021) 38:125–38. 10.1111/hir.12295 31995273

[21] Zhong D Luo S Zheng L Zhang Y Jin R. Epilepsy occurrence and circadian rhythm: a bibliometrics study and visualization analysis via citespace. Front Neurol. (2020) 11:984. 10.3389/fneur.2020.00984 33250835 PMC7674827

[22] Chen CM. CiteSpace II: Detecting and visualizing emerging trends and transient patterns in scientific literature. J Am Soc Inf Sci Technol. (2006) 57:359–77. 10.1002/asi.20317

[23] Wang S Zhou H Zheng L Zhu W Zhu L Feng D. Global trends in research of macrophages associated with acute lung injury over past 10 years: a bibliometric analysis. Front Immunol. (2021) 12:669539. 10.3389/fimmu.2021.669539 34093568 PMC8173163

[24] Merigo JM Pedrycz W Weber R de la Sotta C. Fifty years of information sciences: a bibliometric overview. Inf Sci. (2018) 432:245–68. 10.1016/j.ins.2017.11.054

[25] Koseoglu MA Rahimi R Okumus F Liu J. Bibliometric studies in tourism. Ann Tourism Res. (2016) 61:180–98. 10.1016/j.annals.2016.10.006

[26] Hirsch JE. An index to quantify an individual's scientific research output. Proc Natl Acad Sci USA. (2005) 102:16569–72. 10.1073/pnas.0507655102 16275915 PMC1283832

[27] Molinari J-F Molinari A. A new methodology for ranking scientific institutions. Scientometrics. (2008) 75:163–74. 10.1007/s11192-007-1853-2

[28] Zhou J Li JQ Zhang JG Geng B Chen Y Zhou XB. The relationship between endorsing reporting guidelines or trial registration and the impact factor or total citations in surgical journals. PEER J. (2022) 10:1–15. 10.7717/peerj.12837 35127293 PMC8796708

[29] Schetter CD Tanner L. Anxiety, depression and stress in pregnancy: implications for mothers, children, research, and practice. Curr Opin Psychiatry. (2012) 25:141–8. 10.1097/YCO.0b013e3283503680 22262028 PMC4447112

[30] Harris A Seckl J. Glucocorticoids, prenatal stress and the programming of disease. Horm Behav. (2011) 59:279–89. 10.1016/j.yhbeh.2010.06.007 20591431

[31] Biaggi A Conroy S Pawlby S Pariante CM. Identifying the women at risk of antenatal anxiety and depression: a systematic review. J Affect Disord. (2016) 191:62–77. 10.1016/j.jad.2015.11.014 26650969 PMC4879174

[32] Howard LM Molyneaux E Dennis C-L Rochat T Stein A Milgrom J. Non-psychotic mental disorders in the perinatal period. Lancet. (2014) 384:1775–88. 10.1016/S0140-6736(14)61276-9 25455248

[33] Schetter CD. Psychological Science on Pregnancy: Stress Processes, Biopsychosocial Models, Emerging Research Issues. in: Fiske ST. Schacter DL, Taylor SE. (Eds.), Annual Review of Psychology, Vol 62, 2011, pp. 531-558. 10.1146/annurev.psych.031809.130727 21126184

[34] Brown AS The The environment and susceptibility to schizophrenia. Prog Neurobiol. (2011) 93:23–58. 10.1016/j.pneurobio.2010.09.003 20955757 PMC3521525

[35] Glover V. Maternal depression, anxiety and stress during pregnancy and child outcome; what needs to be done. Best Pract Res Clin Obstet Gynaecol. (2014) 28:25–35. 10.1016/j.bpobgyn.2013.08.017 24090740

[36] Malkova NV Yu CZ Hsiao EY Moore MJ Patterson PH. Maternal immune activation yields offspring displaying mouse versions of the three core symptoms of autism. Brain Behav Immun. (2012) 26:607–16. 10.1016/j.bbi.2012.01.011 22310922 PMC3322300

[37] Buss C Davis EP Shahbaba B Pruessner JC Head K Sandman CA. Maternal cortisol over the course of pregnancy and subsequent child amygdala and hippocampus volumes and affective problems. Proc Natl Acad Sci USA. (2012) 109:E1312–9. 10.1073/pnas.1201295109 22529357 PMC3356611

[38] Castañeda-Reyes ED Perea-Flores MJ Davila-Ortiz G Lee Y Gonzalez de Mejia E. Development, characterization and use of liposomes as amphipathic transporters of bioactive compounds for melanoma treatment and reduction of skin inflammation: a review. Int J Nanomedicine. (2020) 15:7627–50. 10.2147/IJN.S263516 33116492 PMC7549499

[39] Chawla NV Davis DA. Bringing big data to personalized healthcare: a patient-centered framework. J Gen Intern Med. (2013) 28:S660–5. 10.1007/s11606-013-2455-8 23797912 PMC3744281

[40] Rodgers AB Morgan CP Bronson SL Revello S Bale TL. Paternal stress exposure alters sperm MicroRNA content and reprograms offspring HPA stress axis regulation. J Neurosci. (2013) 33:9003–12. 10.1523/JNEUROSCI.0914-13.2013 23699511 PMC3712504

[41] Kalra S Einarson A Karaskov T Van Uum S Koren G. The relationship between stress and hair cortisol in healthy pregnant women. Clin Invest Med. (2007) 30:E103–7. 10.25011/cim.v30i2.986 17716540

[42] Hoffman MC Mazzoni SE Wagner BD Laudenslager ML Ross RG. Measures of maternal stress and mood in relation to preterm birth. Obstet Gynecol. (2016) 127:545–52. 10.1097/AOG.0000000000001287 26855101 PMC4764470

[43] Cao-Lei L de Rooij SR King S Matthews SG Metz GAS Roseboom TJ. Prenatal stress and epigenetics. Neurosci Biobehav Rev. (2020) 117:198–210. 10.1016/j.neubiorev.2017.05.016 28528960

[44] Cunningham AM Walker DM Nestler EJ. Paternal transgenerational epigenetic mechanisms mediating stress phenotypes of offspring. Eur J Neurosci. (2021) 53:271–80. 10.1111/ejn.14582 31549423 PMC7085959

[45] Kaali S Jack DW Delimini R Hu LS Burkart K Opoku-Mensah J. Prenatal household air pollution alters cord blood mononuclear cell mitochondrial DNA copy number: sex-specific associations. Int J Environ Res Public Health. (2019) 16:26. 10.3390/ijerph16010026 30583542 PMC6338880

[46] Zhou GY He TK Huang H Feng FF Liu XX Li ZY. Prenatal ambient air pollution exposure and SOD2 promoter methylation in maternal and cord blood. Ecotoxicol Environ Saf. (2019) 181:428–34. 10.1016/j.ecoenv.2019.06.039 31220783

[47] Liu R DeSerisy M Fox NA Herbstman JB Rauh VA Beebe B. Prenatal exposure to air pollution and maternal stress predict infant individual differences in reactivity and regulation and socioemotional development. J Child Psychol Psychiatry. (2022). 10.1111/jcpp.13581 35174891 PMC9381652

[48] Rosa MJ Just AC Guerra MS Kloog I Hsu HHL Brennan KJ. Identifying sensitive windows for prenatal particulate air pollution exposure and mitochondrial DNA content in cord blood. Environ Int. (2017) 98:198–203. 10.1016/j.envint.2016.11.007 27843010 PMC5139686

[49] Keshavarzi F Farnia V Yazdchi K Najafi F Brand S Bajoghli H. Effect of maternal anxiety on maternal serum and fetal cord blood cortisol. Asia Pac Psychiatry. (2014) 6:435–9. 10.1111/appy.12125 24664930

